# Roles of arbuscular mycorrhizal fungi in plant growth and disease management for sustainable agriculture

**DOI:** 10.3389/fmicb.2025.1616273

**Published:** 2025-07-23

**Authors:** Muhammad Umer, Naureen Anwar, Mustansar Mubeen, Yun Li, Amjad Ali, Mohammed O. Alshaharni, Pingwu Liu

**Affiliations:** ^1^School of Breeding and Multiplication (Sanya Institute of Breeding and Multiplication), College of Tropical Agriculture and Forestry, Hainan University, Sanya, China; ^2^Department of Biological Sciences, Faculty of Science and Technology, Virtual University, Lahore, Pakistan; ^3^Department of Plant Pathology, College of Agriculture, University of Sargodha, Sargodha, Pakistan; ^4^College of Agriculture, Guangxi University, Nanning, China; ^5^Department of Plant Protection, Faculty of Agricultural Sciences and Technologies, Sivas University of Science and Technology, Sivas, Türkiye; ^6^Department of Biology, College of Science, King Khalid University, Abha, Saudi Arabia

**Keywords:** arbuscular mycorrhizae, plant disease management, induced systemic resistance, sustainable crop production, biotic and abiotic stress mitigation

## Abstract

Arbuscular mycorrhizal fungi (AMF) are the basis symbionts in terrestrial ecosystems, profoundly influencing plant development, nutrient acquisition, and resilience to biotic and abiotic stresses. This review synthesizes current systematic understandings of AMF-mediated augmentation of plant growth and disease resistance, with a particular emphasis on their role in sustainable crop production. AMF improves host plant performance through enhanced phosphorus, nitrogen, and water uptake via extensive extraradical hyphal networks. Moreover, AMF colonization modulates phytohormonal signaling pathways, including salicylic acid, jasmonic acid, abscisic acid, and nitric oxide, priming SR and upregulating defense-related gene expression. Increased biosynthesis of secondary metabolites, reinforcement of cell walls, and activation of antioxidant enzyme systems often accompany these responses. AMF also engage in synergistic interactions with rhizosphere microbiota such as *Trichoderma, Pseudomonas*, and *Bacillus*, enhancing their collective biocontrol efficacy against a broad spectrum of soil-borne pathogens, including fungi, bacteria, and nematodes. Through modulation of root exudates, glomalin-mediated soil aggregation, and microbiome restructuring, AMF contributes to the establishment of disease-suppressive soils. Genomic and transcriptomic studies have elucidated key components of the common symbiosis-signaling pathway, supporting AMF-host specificity and functional outcomes. AMF is a promising biotechnological tool for integrated pest, disease, and nutrient management. Advancing their application in field settings requires targeted research on strain-host-environment interactions, formulation technologies, and long-term ecosystem impacts, aligning AMF-based strategies with the goals of resilient and sustainable agriculture.

## 1 Introduction

Fungi have developed numerous strategies for plant colonization, ranging from beneficial to fatal for the host. Fungi are perhaps the most complex group of economically and ecologically significant threats in terms of plant pathogens. Fungal infections can cause a wide range of symptoms. Today, ~19,000 fungi are globally recognized as causing crop plant diseases. Fungi can be dormant under unfavorable environmental conditions, even when found on live or dead plant tissues. Certain fungi can develop in host plant tissue and be dispersed through the soil, water, wind, and insects to other crop-growing areas (Jain et al., 2019). However, fungi can be mutualistic or pathogenic; a mutualistic relationship with a host involves growth promotion and development, and mycorrhizae form a reciprocal relationship with host root systems. However, pathogenic fungi cause diseases like anthracnose, rusts, smuts, leaf spot, blight, wilt, gall, scab, root rot, damping-off, mildew, canker, and dieback. These fungal diseases contribute to significant yield loss, commercial crop loss, and decreased crop quality (Iqbal et al., 2018). Rapidly recognizing fungal disease symptoms is an efficient strategy for controlling and preventing the spread of fungal diseases. The timely detection and identification of fungal symptoms are crucial for effective management of plant diseases. The process of crop disease management involves assessing the adverse effects of pathogens on crop yield (Baiyee et al., 2019). People rely heavily on consistent and stable farm production, but fungal diseases can pose significant threats to food safety. To ensure the overall health of plants and crops globally, it is necessary to control plant diseases. To date, numerous methods have been developed to protect plants from diseases. Rather than implementing new and improved agricultural procedures, most farmers have primarily focused on pesticides for the past few centuries (Chen et al., 2023). However, numerous advances in cultivation science have occurred over the last century. Due to the extensive use of fungi, bacteria, nematodes, and other pathogens, as well as the use of chemical pesticides in agriculture, ecosystems are becoming increasingly resistant to pesticides (Mubeen et al., 2023). Additionally, a growing number of their natural enemies have been eliminated, leading to an increase in pests and diseases (Sanchez-Bayo, 2021). Chemical pesticides contaminate the soil, water, and air simultaneously, harming the environment and the organisms in the food chain, including insects, and impairing human health (Zhou et al., 2025b). Pesticide and fertilizer-related food safety issues, as well as the ongoing development in people's living standards, have drawn considerable attention (Razak and Gange, 2023). Thus, one of the primary areas of interest for environmental scientists and plant pathologists is the pursuit of eco-friendly technologies to manage plant diseases and insect pests (Begum et al., 2019). One method that has drawn considerable interest is biological control (Van Driesche et al., 2010) due to its outstanding efficiency, low consumption, environmental safety, and diverse applications. Consequently, soil scientists, plant pathologists, and ecologists have extensively investigated it (Prospero et al., 2021). Due to increasing health concerns, an innovative disease management method, biological management, has been implemented, and many microorganisms help keep plant diseases in check (Aria et al., 2025). The use of this method has been revived for the first time in many years due to its minimal environmental and health risks to humans. Soil-borne fungi, known as AMF, can significantly increase plant resilience to various abiotic stressors and nutrient uptake (Sun et al., 2018; Mehmood et al., 2022). Arbuscular mycorrhizal fungi (AMF) is classified into Glomerales, Archaeosporales, Paraglomerales, and Diversisporales. Within these 4 orders, 25 genera are located in the subphylum Glomeromycotina of the phylum Mucoromycota, which encompasses the majority of AMF species (spp.) (Redecker et al., 2013; Goss et al., 2017). It is obligate biotrophs that consume photosynthetic plant products and lipids to complete their life cycle. AMF-induced growth enhances the absorption of water and mineral nutrients from the surrounding soil while safeguarding plants against fungal infections (Ahammed et al., 2023). Therefore, AMFs are vital endosymbionts that influence plant productivity and ecosystem functioning. It is essential to improve crops sustainably (Gianinazzi et al., 2010; Chaudhary et al., 2025). AMF releases hyphal chemicals into the soil to control the hyphosphere that different microorganisms have invaded. The rhizosphere and bulk soil have distinct microbial compositions compared to the hyphosphere (Wang et al., 2025). The shift in the microbiome has an impact on nutrient cycling in the hyphosphere. The organic nutrition cycle is influenced by variations in microbial function, making the hyphosphere a unique and vital functional zone in ecosystems. AMF forms a symbiotic relationship with nearly two-thirds of terrestrial plants, providing them with essential nutrients and supporting their growth. Particular microorganisms are attracted to their hyphosphere by AMF hyphae, the small area of soil that is impacted by hyphal exudates (Wang et al., 2024). It molds this alleged second DNA of AMF, notably assisting in the turnover and mobilization of nutrients. Beneficial interactions between microbes and plants are a natural phenomenon, and there is ample evidence of the potential advantages these interactions offer for plant development and health. Typically, in controlled laboratory settings, some of the mechanisms underlying these advantages have been elucidated (Gruden et al., 2020). AMF establishes intimate mutualistic associations with the roots of most vegetable crops and more than 70% of terrestrial plant spp. (Pozo De La Hoz et al., 2021). AMF induces MIR against various foliar and root diseases and pests, and AMF can also boost resistance or tolerance in plants to biotic stressors (Abarca et al., 2024). It is acknowledged that plants regulate the degree of fungal colonization in response to their requirements and the surrounding environment (Pozo et al., 2015). Consequently, understanding how AMF symbiosis is regulated and the advantages it offers under certain circumstances requires an understanding of its context dependency. Systemic resistance (SR) induced by AMF has been shown in interactions with several pathogens and might be reflected in the systemic autoregulation of mycorrhizal colonization. It has been hypothesized that plants utilize the autoregulation mechanism as a preventative measure against further mycorrhizal colonization, while simultaneously defending against pathogens (Fiorilli et al., 2024). The rhizosphere microbiome is primarily shaped by host resistance, while the microbiomes of the roots have been found to be significantly influenced by pathogenic fungal infections. Fungal networks in the roots are significantly impacted by plant disease and host resistance, as well as a few spp. predominate in the communities from the healthy plants.

## 2 Role of AMF in enhancing plant growth and stress resistance

The symbiotic relationship between AMF and plants was documented 400 million years ago (Table 1) (Mythili et al., 2025). These connections are formed by a series of biological processes, resulting in numerous advantageous impacts on natural ecosystems and agricultural biotas (Van Der Heijden et al., 2015). The symbiotic relationship of AMF exemplifies a mutualistic interaction that can influence plant growth and development. The mycelial network of fungi spreads beneath plant roots, facilitating the absorption of nutrients that are normally unavailable (Ahmed et al., 2025). The fungal mycelium infuses the roots of numerous plant spp., forming a common mycorrhizal network (CMN) (Figueiredo et al., 2021), and it is considered a central component of the terrestrial ecosystem, intensely affecting several plant communities, principally invasive spp., and permitting the AMF-mediated transfer of nitrogen (N) and phosphorous (P) to plants (Begum et al., 2019). AMF developed synergistic interactions with plants by colonizing their root systems, contributing to enhanced water uptake and nutrient absorption, as well as increased resistance against biotic and abiotic stresses (Boyno et al., 2023). It can improve soil structure and stimulate plant growth in standard and complex conditions. AMF enhances the tolerance of plants in saline soils by enriching soil structure and supporting various plant mechanisms, including the uptake of water and nutrients, antioxidant defense systems, photosynthesis, and the production of secondary metabolites (SMs) (Boorboori and Lackoova, 2024). It is considered a natural biofertilizer that supplies the host with water, nutrients, and pathogen defense in return for photosynthetic byproducts. Therefore, AMF are important biotic components of soil, and their absence or shortage can result in reduced ecosystem competence. Sustainable agriculture can be achieved by reinstating the natural abundance of AMF, as it is a practical alternative to traditional chemical fertilization. The primary method to achieve this goal involves the direct reintroduction of AMF propagules into the specified soil. AMF has no specific host or niche preferences, signifying their potential role in agriculture across a variety of environmental settings (Berruti et al., 2015). AMF inoculation has the potential to maintain and stabilize soil organic carbon (SOC) by promoting the growth of fungal communities. In N-scarce soils, AMF also simultaneously reduce microbial extracellular enzyme activity. AMF contributes to the enrichment of a persistent carbon sink in drylands through its selective influence on SOC components as a rhizospheric carbon engineer (Li et al., 2025). AMF inoculation significantly increased the abundance and diversity of the rhizosphere fungal community, with a more complex co-occurrence network. The abundance and diversity of the rhizosphere bacterial community were reduced significantly (Chang et al., 2021). AMF symbiosis significantly increased the allocation of photosynthetic carbon to the roots and rhizosphere soils of maize plants. AMF inoculation promoted the levels of macro-aggregates in the soil and microbial biomass carbon in low SOC conditions and increased the formation of soil aggregates, as well as the chemical composition of SOC (Li et al., 2024a). The influence of AMF on SOC sequestration is significant, as it alters the quantity and quality of carbon incorporated and the processes regulating its transformation and storage (Liu and Chen, 2024). Glomalin-related soil protein (GRSP) is mainly produced through the decomposition of AMF mycelium and is a varied assortment of plentiful extracellular proteins along with other components (Ling et al., 2025). Elevated concentrations of GRSP in soils signify increased soil aggregate stability and improved long-term SOC and N sequestration. Meanwhile, extended AMF inoculation reduces soil N stocks and inhibits microbial hydrolase synthesis for carbon substrates (Li et al., 2025). GRSP provides a significant source of many macro and micro-elements, such as C, H, O, S, K, P, Ca, Si, Fe, Cu, and Mg, which are vital for plant development and aid in the immobilization of heavy metal pollutants in soils and sediments (Ji et al., 2025). Glomalin protects soil from dehydration by improving its water retention ability. It consists of 30–40% carbon and related chemicals (Sharma et al., 2017). AMF, as natural root symbionts, provide essential inorganic nutrients to host plants, hence improving growth and yield under both stressed and unstressed situations (Begum et al., 2019). The inoculation of AMF influences growth functions, including stomatal conductance, leaf water potential, relative water content (RWC), PSII efficiency, and CO_2_ assimilation. AMF enhances nutrient absorption, greatly increasing plant resilience to drought, salinity, and heavy metal stress through optimizing water usage efficiency and the modulation of physiological metabolic processes. Additionally, AMF stimulate the plant immune system, augmenting resistance to soil-borne diseases and nematodes, and improving crop safety and quality (Nie et al., 2024). Furthermore, the inoculation of AMF augments water and dry matter absorption, improving plant resilience to stressors such as salinity and desiccation. Employing AMF for plant development across diverse biological environments can significantly enhance organic cultivation, aiming to optimize yield and foster growth.

**Table 1:** The impact of AMF inoculation on agrosystem services.

**AMF symbionts**	**Agrosystem service**	**Crop tested**	**Conditions**	**References**
*Glomus coronatum, G*. *intraradices*, and *G*. *mosseae*	Tackling wildflowers and optimizing P absorption	Sunflower	Greenhouse	(Fuentes-Quiroz et al., 2022)
*Rhizophagus irregularis* and *Funneliformis mosseae*	Enhanced plant mass and superior grain characteristics.	Chickpea	Agriculture land	(Garg and Cheema, 2021)
*G*. *fasciculatum, G*. *etunicatum, G*. *clarum*, and *G*. *Versiforme*	Superior growth attributes.	Long pepper	Center, garden, and field	(Diagne et al., 2020)
Native AMF	Supplemented absorption of Mg, Ca, and K	Maize	Arena	(Wahid et al., 2020)
A mixture of *Gigaspora clarum, G*. *margarita*, and *Acaulospora* sp.	Preservation against Zn and Cu toxicity.	Coffee	Greenhouse	(Andrade et al., 2010)
*G*. *viscosum, R*. *intraradices, G*. *aggregatum, G*. *claroideum*, and *G*. *etunicatum*	Optimal crop growth, output, and grain attributes.	Maize	Arena	(Emmanuel and Babalola, 2020)
*G*. *intraradices*	Boosted fruit attributes.	Strawberry	Glasshouse	(Fuentes-Quiroz et al., 2022)
*G*. *etunicatum*	Elevated growth under salinity	Soybean	Greenhouse	(Igiehon et al., 2021)
*G*. *clarum*	Supplemented fruit production under salinity	Pepper	Glasshouse	(Sałata and Buczkowska, 2020)
*G*. *mosseae*	Dealing with *Meloidogyne incognita*	Tomato	Greenhouse	(Wang et al., 2020)
Native AMF inoculum (consortium)	Adapting plant responses to zinc	Tomato	Environment chamber	(Boyno et al., 2022)
*G*. *mosseae*	Boosted growth cycle, flowering stage, and fruit formation.	Tomato	Growth chamber	(Fayaz and Zahedi, 2021)
*G*. *intraradices*	Advanced harvest results.	Onion and Tomato	Field	(Emmanuel and Babalola, 2020)
*G*. *mosseae, G*. *deserticola*, and *A*. *laevis*	Elevated nutritional content.	Yam	Glasshouse	(Ivanov et al., 2019)
*G*. *mosseae* and *G*. *etunicatum*	It helps to enhance plant growth, yield, and nutrient absorption.	Brinjal and Wheat	Field	(Alaux, 2020)

## 3 AMF as biocontrol agents against soil-borne plant pathogens

AMFs inhabit the soil and infect plant roots, substantially influencing soil-borne diseases (Cruz and Ishii, 2012; Li et al., 2021b). AMF has been extensively utilized as a biological control strategy against several phytopathogenic fungi (Lin et al., 2021). The biocontrol efficacy of AMF has been documented across various plant spp. and against numerous diseases, predominantly soil-borne fungal pathogens responsible for root rot or wilting. Successful biocontrol has also been documented against aerial infections, including *Alternaria solani* in tomatoes (Harrier and Watson, 2004). AMF has been reported to reduce both necrotrophic and biotrophic diseases directly or indirectly (Schouteden et al., 2015). AMF establish a symbiotic association with plant roots, thereby playing a crucial role in managing soil-borne diseases (Cruz and Ishii, 2012) and are extensively utilized as biocontrol agents against plant pathogenic fungi (Lin et al., 2021). Mycorrhizal cotton plants have shown superior resistance to infection by the pathogen *Thielaviopsis basicola* compared to those with sterile roots. Later studies demonstrated that the generation of chlamydospores by *T*. *basicola* was inversely correlated with the degree of mycorrhizal infection (Thakur et al., 2024). The interaction between AMF and *Rhizobium*, alongside two pathogenic fungi, *Pythium ultimum* and *Phytophthora megasper*, showed that mycorrhizal fungi reduced the occurrence of plant death caused by *P. megasper* (Chou and Schmitthenner, 1974; Ghorui et al., 2024). Compared to the control group without AMF inoculation, the illness index and incidence of *Ralstonia solanacearum* were reduced by 9.7% and 49.8%, respectively, when infected with *G. rhizogenes* and *G. mossie* (Steinkellner et al., 2012). *G*. *asciculatum, G*. *etunicatum, G*. *macrocarpum, G*. *Margarita, G*. *heterogama*, and *G*. *calospora* in AMF can mitigate diseases induced by pathogenic fungi from the genera *Pythium, Phytophthora, Fusarium, Rhizoctonia, Macrophomina, Pyrenochaeta, Thielaviopsis, Phoma, Cylindrocarpum, Ophiobolus*, and *Sclerotium* in barley, peanut, soybean, banana, cotton, kidney bean, onion, tobacco, citrus, peach, poplar, strawberries, red clover, and ginseng (Weng et al., 2022). *G*. *intraradices* inhibited the proliferation of the pathogenic fungus *F. oxysporum*, suggesting that the chemical equilibrium of mycorrhizae suppresses the growth and reproduction of pathogenic fungi (Singh, 2020). Infected peas with *Aphanomyces euteiches* demonstrate that establishing a complete AMF symbiosis is crucial for plant defense against pathogens (Slezack et al., 2000; Wang et al., 2022). *Phytophthora* is a typical pathogenic fungus widely employed in the treatment of plant diseases associated with AMF (Krzyzaniak et al., 2021). The application of P and AMF pre-treatment in tomatoes infected with *G. intraradices* and the pathogen *F. oxysporum* resulted in diminished disease severity. Factors such as the specific plant disease, the interaction between AMF and host plants, the amount and timing of AMF inoculation, and environmental variables (Weng et al., 2022) all affect the effectiveness of AMF in managing plant diseases. *Phytophthora* served as a model pathogenic fungus to elucidate the mechanism of AMF-mediated disease control (Krzyzaniak et al., 2021). The efficacy of *G. intraradices* against *F. oxysporum* can be enhanced by the use of P, thereby reducing disease severity in tomatoes (Steinkellner et al., 2012). The disease control mechanism of AMF is affected by various aspects, including the pathogenic organism, the symbiotic interaction between AMF and the host, the timing and concentration of AMF inoculation, and environmental conditions.

## 4 AMF in managing bacterial and nematode-induced plant diseases

AMF plays a crucial role in regulating bacterial and nematode diseases through diverse molecular mechanisms and signal transduction pathways (Schouteden et al., 2015). SR is induced in host plants by the colonization of AMF through the activation of defense-related genes (DRGs), including those involved in salicylic acid (SA), jasmonic acid (JA), and ethylene (ET) signaling pathways that are fundamental in the resistance against biotrophic and necrotrophic pathogens (Stratton et al., 2022). AMF also regulates the expression of pathogenesis-related proteins and stimulates the activity of antioxidant enzymes, thereby limiting oxidative stress during pathogen attack. AMF also alter root exudation patterns, indirectly inhibiting nematodes and soil-borne pathogens by restructuring the rhizosphere microbiome (Schouteden et al., 2015; Afridi et al., 2024). AMF-mediated suppression of *Meloidogyne incognita* and *R*. *solanacearum* is coupled to the upregulation of defense-related genes (DRGs) such as PR-1, LOX, and PAL and enhanced production of secondary metabolites (SMs), including phenolics and flavonoids (Vos et al., 2013; Zhu et al., 2018). Additionally, pathogen entry is restricted due to AMF-stimulated cell wall modifications and the deposition of callose and lignin (Underwood, 2012). Inoculation of tomato plants with *G*. *intraradices* induced the expression of PR-1 and PR-5 genes, thereby enhancing resistance to *R*. *solanacearum* (Gao et al., 2004). *G*. *mosseae* also enhanced soybean resistance against *Heterodera glycines* by upregulating JA- or ET-regulated defense genes and antioxidant enzyme activity (Guo et al., 2015). In cucumber, AMF colonization led to the upregulation of lignin biosynthesis and SM genes, which limited *M*. *incognita* penetration and gall formation (Schouteden et al., 2015). *R*. *solanacearum* induced bacterial wilt in tomatoes (Yuliar et al., 2015), and it can be controlled with mycorrhizal application. Inoculation of mulberry with *G*. *fasciculatum* or *G*. *mosseae* in combination with 60–90 kg of P per hectare per year reduced the incidence of bacterial blight caused by *P*. *syringae* (Imad Khrieba, 2019). AMF application in grape fields has harmed the population of *P. fluorescens* in the rhizosphere and reduced the likelihood of disease recurrence. *G*. *mosseae* suppressed *P*. *syringae* and safeguarded soybean and apple seedlings, which can be protected by root treatment with AMF against Actinomycetes. *M. incognita* and *M. javanica* can cause total crop failures in tobacco, tomato, sunflower, and pepper, respectively, while AMF symbionts enhance plant tolerance to nematodes (Schouteden et al., 2015). However, it can only inhibit the damage caused by nematodes (Weng et al., 2022). AMF reduces infection and reproduction of root-knot nematodes in crops like tomatoes, bananas, and coffee (Schouteden et al., 2015). *G. mosseae* and *Rhizophagus irregularis* reduce infection in bananas by *Radopholus similis* (Mandou et al., 2023) and controlled *M*. *exigua* in coffee plants (Alban et al., 2013). Soybean cyst nematodes parasitised by AMF and the degree of disease-causing ability in soybeans, oats, cucumbers, cotton, kidney beans, tomatoes, citrus, peach, and alfalfa is decreased (Rodrigues et al., 2021). The inoculation of *G*. *mosy* controlled the *M. incognita* population in tobacco and developed disease resistance against nematodes (Liu et al., 2012). Mycorrhizated plants showed fewer galls on the roots of tomato plants than non-mycorrhizated plants, and the infection rate was significantly reduced. AMF colonization can modify host root exudates (Ma et al., 2022) and enhance the antagonistic rhizosphere environment toward pathogens such as *Pseudomonas syringae* and *Agrobacterium tumefaciens*, in addition to affecting the levels of phenolic acids in cotton root exudates, hence reducing the incidence of cotton Fusarium wilt (Zhang et al., 2012). Furthermore, AMF enhance callose deposition, cell wall fortification, and detoxification of reactive oxygen species, which are essential at the early stages of pathogen invasion (Nath et al., 2016). Subsequent to AMF colonization, it can limit nematode motility and alleviate nematode infestation in tomatoes by affecting the release of root exudates (Yizhu et al., 2020). AMF colonization can improve resistance by modifying host root exudates. The colonization by AMF affects changes in plant root exudates, and these variations in exudates simultaneously influence the growth and development of AMF, and it interacts with others rather than existing independently (Zhang et al., 2024). The total molecular responses attest to the potential of AMF as an efficient biocontrol agent in agricultural practices.

## 5 Symbiotic interactions of AMF with microbiota in plant disease management

The synergistic effect of AMF and *Trichoderma harzianum* is more considerate in the management of severity and incidence of diseases than the use of *T*. *harzianum* and AMF alone, and studies showed combined application in the field of *Solanum lycopersicum* enhanced aboveground biomass by 11.6–69.7% (Weng et al., 2022). Inoculation of *F*. *oxysporum* on tomatoes resulted in a disease incidence rate of 70%. After applying *Acaulospora laevis* and *G*. *mosseae*, the decrease was 20%. However, a 10% reduction was found with the inoculation of *T*. *virid* and AMF (Tanwar et al., 2013). AMF and *Trichoderma* can together prevent the occurrence of disease. However, their different combinations have different control effects on plant diseases. If AMF was individually inoculated against *Cucumis melo* Fusarium wilt, it only reduced disease incidence from 25% to 60% (Martinez-Medina et al., 2010). Furthermore, the same combination of *Trichoderma* and AMF also has varying effects on the different spp. types. The disease control effect of *T*. *harzianum* and *G*. *clarum* for HEL246 (a variety of *Halianthus tuberosus*) was the best. At the same time, AMF alone was the best control for variety JA37 (Sennoi et al., 2013). Synergistic effect of *Pseudomonas* and AMF refining plant disease resistance rather than application individually. The individual application of *G*. *albida, G*. *sinosum*, or *P*. *fluorescens* against the disease induced by *Phaseolus vulgaris* can only reduce the disease by 50.5 to 52.8%, while the combined application shows a reduction from 68.9 to 69.2%. The combined application increases the P and N contents of plants compared to single inoculation (Neeraj and Singh, 2011). It was discovered that a combination inoculation of *G. sinuosum* and *P. fluorescens* was more successful against the diseases caused by *F*. *oxysporum* in tomatoes (Srivastava et al., 2010) and papayas (Hernández-Montiel et al., 2013). However, the synergistic effects of *P*. *fluorescens* and AMF on plants were not all positive. When applied together, *P. fluorescens* + *G. messeae* had a more significant growth-enhancing impact than when applied alone in the absence of pathogenic microorganisms (Behn, 2016). In addition, the combined applications of AMF and *P*. *aeruginosa* manage plant diseases, as do the applications of *P*. *fluorescens* and AMF. *Elaeis guineensis* base rot severity was reduced from 15% to 17% when inoculated with AMF alone (*G*. *clarum* and *G*. *intraradices*). In contrast, if combined with *P*. *aeruginosa*, the reduction of severity was found to be 57–80% (Parvin et al., 2020). The synergistic biocontrol effect of *Bacillus* and AMF on diseases of plant roots is the best control method. The mixed use of *Bacillus subtilis* and *G. mosseae* can decrease the disease severity of tomato fusarium root rot from 85% to 93.4%. In addition, it is involved in plant nourishment (N, potassium (K), P, magnesium, calcium, zinc, and iron), total soluble protein, total soluble sugar, total free amino acid content, and leaf pigment (Cai et al., 2021). Single *B*. *vallismortis* and *G*. *versiforme* can decrease the verticillium wilt index for cotton from 35.7% to 37.7%, respectively. Still, a combined application can reduce the disease by up to 63.3% (Zhang et al., 2012). Additionally, it can be 73.6 to 82.1% effective when applied against *F. oxysporum* (Cai et al., 2021) and 34.1 to 52.1% effective when applied singly (Rashad et al., 2020). *Glomus* can enhance the ability of *B. subtilis* to suppress strawberry Fusarium wilt (Tahmatsidou et al., 2006). Strawberries with a combination inoculation had a 61.7–90.9% increase in fresh weight as compared to a single application (Table 2).

**Table 2:** The impact of AMF inoculation on plant pathogen management.

**AMF strains**	**Host plant**	**Pathogen**	**Growth improvements**	**References**
*Rhizophagus irregularis*	Soybean	*Macrophomina phaseolina*	Enhanced plant ability to stand by activating the immune system and increasing plant biomass	(Vandegrift et al., 2023)
*F. mosseae*	Tomato	*Cladosporium fulvum*	Enhanced plant ability to stand by activating the immune system, increased plant water content, and enhanced seedling weight	(Kumari and Prabina, 2019)
*Glomus* spp.	Pepper	*Pythium aphanidermatum*	Enhanced plant ability to stand by activating the immune system and increasing plant biomass	(Frac et al., 2023)
*Gigaspora margarita* and *G*. *etunicatum*	Sugarcane	*Scutellospora fulgida*	Enhanced plant ability to stand by activating the immune system, raised phenolic and proline content, enhanced antioxidant enzyme activities	(Kumari and Srimeena, 2019)
*R*. *irregularis* and *R*. *fasciculatus*	Tomato and pepper	*F*. *oxysporum*	Improved root systems of plants and reduced membrane damage, enhanced nutrient uptake, and reduced lipid peroxidation	(Aylward et al., 2023)
*Claroideoglomus etunicatum, G. versiforme, F. mosseae*	Prairie Milk-vetch	*Erysiphe pisi*	N, K, and P play essential roles among the crucial nutrients for plants	(Spagnoletti et al., 2020)
*F*. *caledonium*	Tomato	*R*. *solanacearum*	Reduced wilt symptoms, increased phenolic compounds, and defense gene expression	(Li et al., 2021a)
*R*. *irregularis*	Banana	*R*. *solanacearum*	Decreased bacterial wilt incidence, enhanced root architecture, and SR	(Lin et al., 2021)
*G. mosseae*	Tobacco	*R*. *solanacearum*	Induced DRGs and reduced bacterial populations	(Yuan et al., 2016)
*G. mosseae*	Tomato	*M*. *incognita*	Reduced gall formation, enhanced lignin content, and chitinase activity	(Ma et al., 2022)
*G*. mosseae, *Gigaspora gigantea*, and *P*. *fluorescens*	Eggplant	*M*. *javanica*	Reduced root-knot nematode infestation and improved plant growth	(Sharma et al., 2021)
*G*. *mosseae*	Soybean	*H*. *glycines*	Enhanced soybean resistance through the upregulation of JA or ET-regulated defense genes and antioxidant enzyme activity	(Guo et al., 2015)
*G*. *mosseae*	Banana	*M*. *incognita*	Enhancing growth by plant nutrition and suppressing nematode reproduction and galling during the early stages of plant development	(Jaizme-Vega et al., 1997)

## 6 Non-symbiotic interactions of AMF with microbiota in plant disease management

AMF participates in non-symbiotic interactions with soil microbiota, which significantly affect plant disease management. While not characterized by direct symbiotic nutrient exchange, these interactions influence microbial community composition and activity, thereby improving plant resilience to pathogens (Purohit et al., 2024). AMF exudates, including strigolactones and glycoproteins, promote the growth of beneficial rhizobacteria and fungi, thereby enhancing a suppressive soil environment (Ghorui et al., 2024). AMF-induced alterations in the rhizosphere microbiome increase the prevalence of *Pseudomonas* and *Bacillus* spp., which produce antibiotics and siderophores that inhibit soil-borne diseases (Lahlali et al., 2022). Additionally, AMF hyphae create an environment conducive to microbial colonization, enhancing niche competition and reducing pathogen viability (Yuan et al., 2021). Non-symbiotic interactions enhance plant SR by activating DRGs and phytohormone signaling pathways (Mhlongo et al., 2018). Furthermore, AMF-induced changes in soil aggregation and organic matter breakdown impact microbial habitat dynamics, thereby indirectly reducing the proliferation of pathogens (Frey, 2019). Field studies have demonstrated that AMF-associated microbiota minimize the occurrence of Fusarium wilt and Phytophthora root rot, highlighting their potential as biocontrol agents (Kashyap et al., 2024). Utilizing non-symbiotic AMF-microbiota interactions offers a sustainable strategy for integrated disease management, reducing reliance on chemical fungicides and enhancing soil health.

## 7 Expanding research on AMF in plant disease control mechanisms

**Figure 1:**
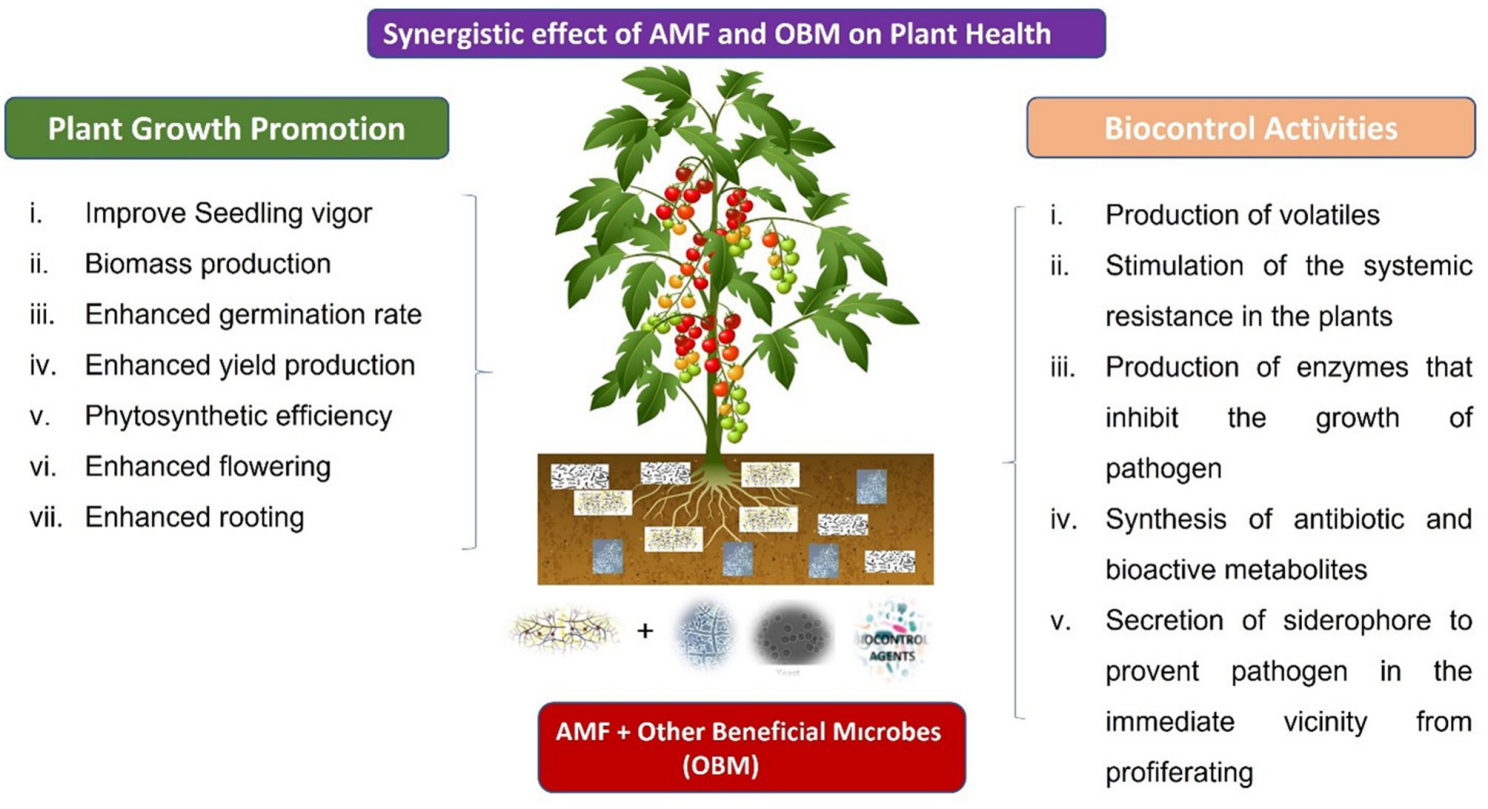
The network highlighted key terms in the article title, abstract, or keywords related to the AMF and their role in biotic stress management. In the network, the same color shows a cluster of interconnected phrases; however, the circle size indicates the number of publications.

The primary mechanisms associated with the research on utilizing AMF in the control of plant diseases include enhancing the micro-environment of the rhizosphere, modifying the morphological structure of plant roots, improving plant nutrition, sustaining the synthesis of SMs, directly competing with pathogenic microorganisms for invasion sites and nutrients, and inducing the formation of plant defense systems and disease resistance (Figure 1) (Tatsumi et al., 2020; Chen et al., 2021).

### 7.1 Structural modifications induced by AMF symbiosis for enhanced plant resistance

**Figure 2:**
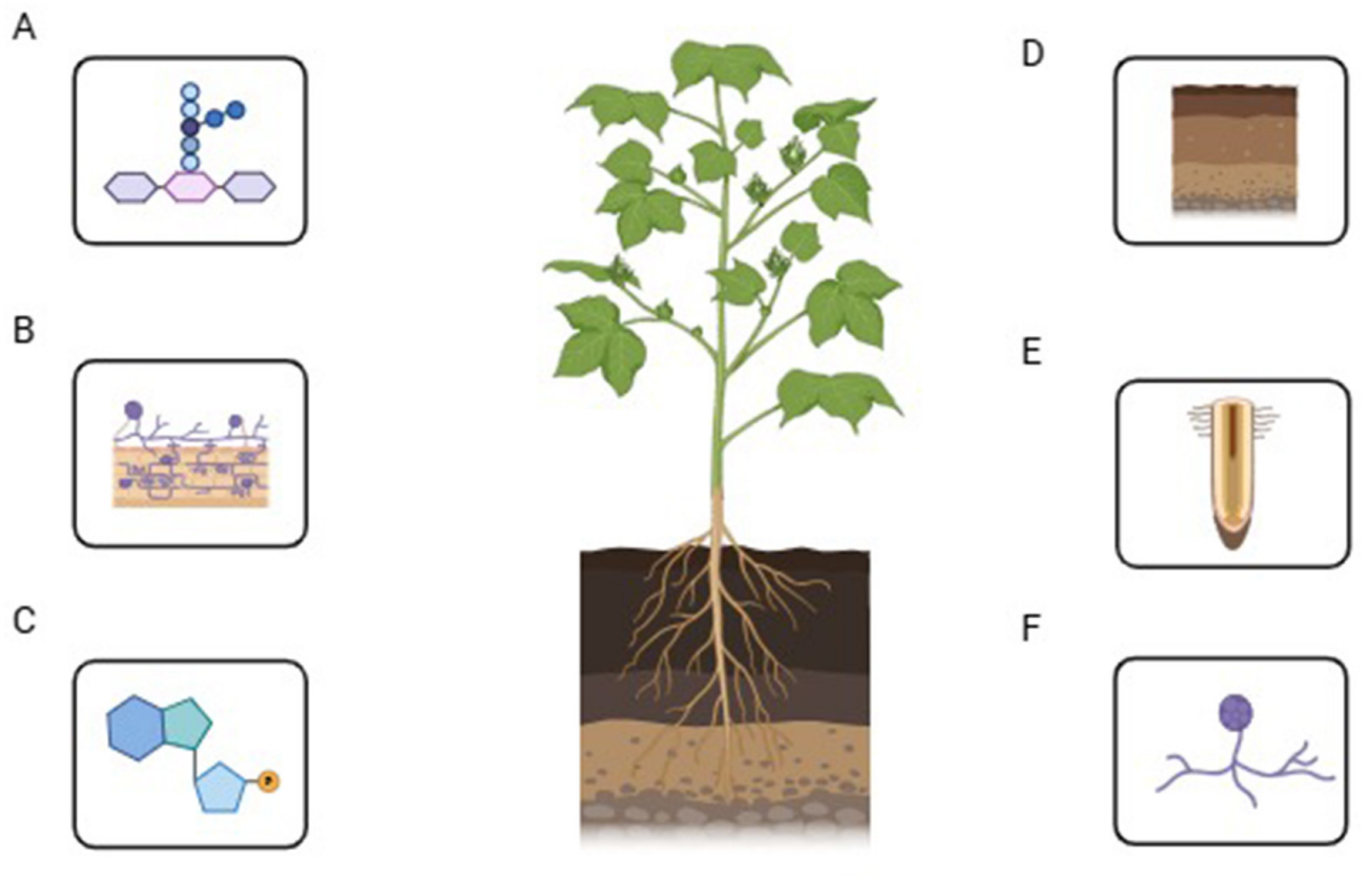
Functional role of AMF within the host roots. **(A)** Role of AMF in regulating the morphology of plant roots; **(B)** Mycelial network and callose formed by AMF infection; **(C)** How the AMF helps the cell wall of the roots of mycorrhizated plants to produce HRGPs and alters the root system; **(D–F)** The symbiotic association of AMF with the plant roots and their impact on root exudate secretion.

In host plants, AMF can lead to the growth, thickening, and branching of the root system, effectively slowing down the virus infecting the roots (Basyal and Emery, 2021; Figure 2A). Symbiotic roots with *G*. *etunicatum* and *G*. *mosseae* of *Gossypium hirsutum* increased root xylem structure due to *Verticillium dahliae* effect, and deformed vessels produced gelatinous substances, shrunken and altered cells, palsied tissue, significant thickening of a cell wall, and deepened color of cells (Weng et al., 2022). The number of vacuoles in cells decreased, the inner folds of mitochondria disappeared, and the root system underwent multiple structural changes, all of which positively improved host resistance against *Verticillium dahliae*. Mycelial network and callose formed by AMF infection induced papillary structure in the root epi and endodermis with the arrangement of non-esterified pectin (Figure 2B), which is an obstacle for the penetration of pathogen in root cell and tissues and AMF alter the anatomy of tomato roots and changed infection kinetics of *Phytophthora* (Pozo et al., 2002). Plants and AMF make fully functional symbiotic interactions by establishing surface contact that initiates nutrient exchange and signal transduction. The symbiotic interface is defined as a molecular exchange between plants and AMF cytoplasm via cell walls and plasma membranes (Balestrini and Bonfante, 2014). AMF stimulated the production of hydroxyproline-rich glycoproteins (HRGPs) in mycorrhizal plants (Balestrini and Bonfante, 2014). HRGPs are sugar-containing linear proteins embedded in the plant cell wall. When pathogens attack, these proteins reinforce the cell wall, reducing the breakdown caused by pathogen-secreted enzymes such as proteases, hemicellulases, and cellulases. Additionally, HRGPs function like lectins, acting as adhesive molecules that trap and immobilize invading pathogens, thereby preventing their further penetration into plant cells. AMF also modifies the root system architecture, improving plant resistance against infections (Figure 2C). Some spp. of *Glomus*, extra-root hyphae, the cell wall of spores, and the germ tube inner wall contain β-1, 3-glucan, while β-1, 3-glucan is not present in the cell wall of *Gigaspora* or *Scutellospora* spp. (Ma et al., 2021a). It serves as a structural component and provides a defensive barrier against pathogens. Employing AMF as a biocontrol agent can modify the root's anatomical structure and enhance naturally occurring defensive compounds, thereby boosting the innate resistance in plants to diseases and pests (Silvestri et al., 2020).

### 7.2 Enhancement of water and nutrient uptake by AMF for improved plant resistance

AMF enhances the absorption of water and vital mineral elements by plants. Research indicates that AMF establishes a vast mycelial network in the soil, with their extra-radical hyphae interlacing, so considerably enhancing the root system. This network improves the uptake of water, nitrates, phosphates, and other essential nutrients, benefiting numerous plants concurrently. Furthermore, AMF enables the movement of water and nutrients across plants, establishing an alternative and highly efficient mechanism for resource acquisition (De La Rosa-Mera et al., 2011). The 14C labeling technology was used in 1993, and it was found that a very minute quantity of 14C was released around the citrus mycorrhizal roots (Eissenstat, 1993). It was because of the competition between pathogens and AMF for photosynthetic products secreted by host plant roots. AMF utilizes photosynthetic product materials first, which diminishes the pathogen's acquisition chance, thereby reducing its ability to grow and reproduce (Kuila and Ghosh, 2022). By enhancing nutrient and water uptake, mycorrhizae mitigate root damage caused by pathogens, thereby reducing harm and improving plant resilience (Ma et al., 2021a). Tomato inoculation under *F*. *oxysporum* stress increases chlorophyll, soluble sugars, branching, and leaf development while improving nutrient absorption (P, N, K, Mn, Zn, Ca), thereby enhancing disease and pest resistance (Liang et al., 2021).

### 7.3 Mechanism of AMF-induced production of SMs

The presence of SM compounds in AM-colonized plants enhanced the expression of pathogenesis-related genes and increased the production of volatile compounds, including aldehydes, ethers, and alcohols, across different plant parts (Quaglia et al., 2012). Multiple enzymes facilitate the production of these metabolites. Lipoxygenases serve as critical signaling molecules that trigger defense responses in crop plants (Singh et al., 2022). The mechanisms underlying the alteration of the number of SMs remain unknown. Colonization by AMF leads to increased concentrations of phenolic, terpene, and nitrogenous compounds in shoot and root plant tissues (Kumar et al., 2021). The results of the study indicate that symbiotic colonization by AMF improves the nutritional value of the plants due to increased P and N absorption from the soil. The infection of host plants with AMF generally promotes the uptake of P, thereby enhancing the nutritional value and the levels of SMs as well as phytochemicals in the plants (Selwal et al., 2023; Wu et al., 2024). The alteration in SMs production may be an outcome of the introduction of changes by AMF in phytohormone pathway-associated plant pathways (Amani Machiani et al., 2022). The pathways involve those participating in gibberellin acid (GA), abscisic acid (ABA), brassinosteroids (BR), auxin (IAA), SA, JA, cytokinin (CK), and ET. Besides that, the symbiosis enhances the plant's defense mechanism (Schmitz and Harrison, 2014). Cucumber plants inoculated with *Gigaspora terrestris* contained high levels of IAA, zeatin, and GA. The high IAA levels enhance *Rhizoctonia solani* resistance by activating the defense mechanisms in plants against pathogen attack (Metwally and Al-Amri, 2020). AMF enhanced GA gene expression in *Medicago truncatula* (Ortu et al., 2012), JA and GA enhanced the concentration of terpenoid components through the induction of glandular trichomes development and improved expression of sesquiterpenoid biosynthetic genes (Singh and Sharma, 2015). The signaling molecules involved in AMF and host-plant symbiosis have the potential to modulate the content of SMs in plants. A symbiosis between *Trifolium repens* and *G*. *mosseae* enhances the content of signaling molecules such as salicylic acid, nitric oxide, and hydrogen peroxide, which in turn elevates the activity of enzymes related to phenolic biosynthesis (Zhang et al., 2013). Mycorrhizal plants have higher phytohormone levels (ABA, IAA, CK, GA, and ET) in leaves and stems compared to non-mycorrhizal ones. Induction of those participating in the phytohormone pathway, i.e., AMF, directly influences plant growth and indirectly affects resistance. Under stress, these hormones can alter the expression of genes and regulate gene synthesis, thereby enhancing the adaptability of plants (Weng et al., 2022).

### 7.4 AMF-induced production of SMs for plant disease resistance

A key mechanism by which AMF enhances plant disease resistance is the regulation of SMs production. This occurs as the mycorrhizal symbionts influence the physiological metabolism of plants, altering both the quantity and diversity of these defensive compounds (French, 2017). SMs are advantageous for plants because they help them combat harmful conditions caused by infection. A class of resistant substances known as phytoprotectins is initiated in response to pathogenic infection. The rate and amount of accumulation of these compounds are connected to the ability of plants to resist diseases (Monther Mohumad, 2012). The accumulation of phytophanins serves as a barrier around infected cells to prevent the spread of auxiliary pathogens (Jaiti et al., 2008). *G. mosseae* enhances phytotoxin production in response to infection, boosting plant resistance. Additionally, inoculation of *G. intraradices* on cucumber roots promotes callose deposition, which helps protect against the toxic effects of *Colletotrichum orbiculare* (Bais et al., 2006). AMF infection significantly increases the vinblastine in *Catharanthus roseus* leaves and protects them against biotic stresses (Martinez-Medina et al., 2010). The compounds belong to the phenolic family, e.g., phenolic carboxylic acids and flavonoids act as signaling molecules in the defense system (Monther Mohumad, 2012). Flavonoids were found to attract AMF toward plants and expand the symbiotic relationship between AMF and plants (Pei et al., 2020). In the roots of *Gossypium hirsutum*, upon infection of AMF, the production of phenolic substances escalates, and resistance toward *Verticillium dahliae* rises (Lioussanne et al., 2008). *G*. *mosseae* was found to stimulate the higher production of ascorbic acid and polyphenol content in strawberries, while also reducing the severity and disease incidence of *C*. *gloeosporioides* and *F*. *oxysporum* (Chandanie et al., 2009). The use of the root-splitting technique in tomato plants, SR against *Ralstonia solanacearum*, can be induced through *G*. *versiforme* (Zhu and Yao, 2004). In both uninfected and infected roots, the production of phenolic compounds is significantly increased (Weng et al., 2022). Therefore, plant resistance is based on the enhanced production of phenolic compounds. Conversely, AMF inoculated, and AMF uninoculated *Phoenix dactylifera* do not show a rise in the production of phenolic compounds upon infection with *F*. *oxysporum*, accumulation of derivative of hydroxycinnamic acid by mycorrhizated plants shows the ability to halt chlorosis (Jaiti et al., 2008).

### 7.5 AMF influence on root exudates, rhizosphere microbiome, and soil properties

Plants and AMF form a symbiotic association that influences the permeability of root cell membranes, the composition and volume of root exudates, the physical and chemical properties of the rhizosphere, the structural makeup of microbial communities, and the overall microbial density within the rhizosphere (Figures 2D–F). Extra-root hyphae of mycorrhizae can pierce from the minute pores present between soil particles and mycorrhizal secretions, e.g., organic acids, Glomus-associated protein (GRSP), and polyamines involved in the soil particles adhesion, stimulate the soil aggregation, mend soil pH, aeration, water permeability, and stability, promote redox potential (Eh), and enhance the growth of plants to resist pathogenic attack (Tatsumi et al., 2020). Reproduction, growth, and development of soil-borne fungi, bacteria, and nematodes are directly affected by the secretion of root exudates, which stimulate the growth of AMF and plant symbiotic relationships (Ghorui et al., 2024). This symbiotic relationship also affected the microbial community in terms of spatial distribution, nature, quantity, structure, and variation. Nematode invasion in roots is controlled through the secretion of root exudates, which paralyze nematodes with AMF infection in tomatoes (Lone et al., 2024), deter the *Phytophthora nicotianae* zoospores and limit their access to roots (Lioussanne et al., 2008). AMF can form symbiotic relationships with beneficial soil microbes, creating a synergistic effect. This partnership enhances the presence of advantageous microorganisms in the rhizosphere, particularly those that suppress soil-borne pathogens. Trichoderma, Gliocladium, Streptomyces, various antagonistic fungi, Actinomycetes, phosphate-solubilising bacteria, N-fixing bacteria, and plant growth-promoting rhizobacteria (PGPR) (Miransari et al., 2014). These beneficial microorganisms enhance plant disease resistance by decreasing pathogen populations and minimizing the risk of harmful bacterial infections. Furthermore, PGPR can enhance the symbiotic relationship between AMF and plants.

### 7.6 AMF and soil-borne pathogen competition in the rhizosphere

The ecological habitat and intrusion site shared by soil-borne pathogens and the biotrophic symbiotic microbes (e.g., AMF) in the soil rhizosphere are frequently the same. As a result, under the natural environment conditions, pathogens and AMF must interact in their primary biocontrol function to decrease the initial infection and re-infection of root epidermal pathogens in a spatially competitive manner. *G*. *moshe infection* was used to reduce the incidence of *Phytophthora nicotianae* disease, and it cannot infiltrate arbuscular cells in nearby uninfected root systems. In mycorrhizated plants, mycorrhizated roots and nearby non-mycorrhizated roots had a comparatively low population of *H. glycines* (Weng et al., 2022). Competition was seen in inoculated pathogenic bacteria, and AMF was aimed at *Aquilaria agallocha* infection sites (Tabin et al., 2009). The plants of *A*. *agallocha* mycorrhizated with *G*. *fasciculatum* significantly constrain the damping-off symptoms and morbidity index of the root tissue developed by *Pythium aphanidermatum* (Zhou et al., 2020). AMF plays a vital role as a parasite of nematodes, and its hyphae, vesicles, and arbuscular incursion are found in nematode galls such as *G. polygamyces*, which is a parasite of *H. glycines* and induces infection in their eggs (Keshari et al., 2024). Chlamydospores produced by AMF can colonize the cysts of soybean cyst nematodes, and it is a visible indication that AMF is a parasite of nematodes (Vos et al., 2013; Keshari et al., 2024).

## 8 Mechanisms of host defense activation by AMF

### 8.1 Role of AMF-induced signaling substances and phytohormones in plant defense

Signal molecules called phytohormones have the potential to be crucial for the functional regulation of the growth, development, and environmental adaptability of plants. Developing the symbiotic relationship between plants and AMF initiates the synthesis of hormones through plants, or AMF can directly produce hormones (Schmitz and Harrison, 2014). AMF initiates the process of synthesis of different signaling substances, for example, JA, nitric oxide (NO), ET, SA, ABA, hydrogen peroxide (H_2_O_2_), sugar signal, and Ca^2+^ signal, once a symbiotic relationship of plant and AMF is established (Schmitz and Harrison, 2014). The signaling substances are functional in developing a symbiotic relationship between plants and AMF, which triggers the plant's defense system (Metwally and Al-Amri, 2020). ET and JA were found to resist saprophytic infections, which have been reported to be triggered by ET and JA, and SA has an inhibiting impact. It was studied for biotrophic pathogens. ET and JA play essential roles in systemic acquired resistance (SAR) in plants, as opposed to systemic induced resistance (ISR) after the establishment of pathogenic infection (Hause et al., 2007). NO was recognized as a signaling substance and initiator of plant defense system-related gene expression (Calcagno et al., 2012). AMF symbiosis has a strong affinity with the NO accumulation in plants, and alfalfa showed NO content in roots and leaves is 1.9 and 3.3 times higher, respectively, than in control treatment when inoculated with *G*. *margarita*; it suggested that NO accumulation initiated by AMF symbiosis linked with induced SR (He et al., 2010). *F*. *oxysporum*-infected roots of tomato seedlings were inoculated with *G*. *macrocarpum* and *G*. *polyphylla*. After 20 days, disease severity indexing reduced by 75% and 78%, respectively. AMF-stimulated ISR in plants is primarily due to the signaling substance SA (Dugassa et al., 1996). SA application and the inoculation of *G*. *moses* reduced the degree of wilting and disease severity index of *F*. *oxysporum*-infected tomato plants. Cantaloupe is a phytohormone deceased upon infection with *F. oxysporum*, while inoculation of *G*. *rhizogenes* on infected plants increases the production of phytohormone cantaloupe, stimulates the SA and JA signaling pathways, and enhances resistance in plants (Steinkellner et al., 2012). However, *G. intraradiculae* inoculated *Nicotiana attenuata* showed no appreciable changes in endogenous SA and JA contents while slightly reducing ET content (Kapoor, 2008).

### 8.2 AMF-regulated expression of DRGs in plants

The symbiotic association between plants and AMF enhances pathogen resistance by upregulating DRGs (Kashyap et al., 2024). AMF can also modulate the expression of specific resistance genes in plants, enhancing defense responses against particular diseases (Badrbani et al., 2024). In wheat leaves, the expression of genes was remodeled explicitly after *G*. *mosseae* activated the MIR respons*e* against *Zymoseptoria tritici*, and the rate of foliar protection is 78%. Symbiotic relationship of mycorrhizae with plants before pathogenic infection upregulated the PR1 and Pox genes involved in the process of DRGs. After the establishment of infection, the transcriptome profiling revealed that 5 genes (GST, PAL, PR5, CAD, and CalS) were upregulated along with PR1 and Pox in a biotrophic stage of *Z*. *tritici* in leaves (Allario et al., 2025). In soybean plants infected with *Heterodera glycines*, inoculation with AMF led to upregulation of the DRGs (Chib1 and PAL5). This increased expression was confirmed at the transcriptional level using quantitative reverse transcription PCR (qRT-PCR) and Northern blotting techniques. The activation of these genes contributed to induced resistance against nematodes (Li et al., 2005). The Proteomic profiling depicted that upregulation of the DRGs related to transcription factors (such as WRKY), proteases and kinases receptors, auxins production, and encoding proteins related to disease resistance in response to *F*. *virguliforme* induced infection in mycorrhizated soybean plants. However, primed expression was found for DRGs encoding pleiotropic drug resistance and thaumatin-like protein. PODs and modification of cell wall-related DRGs were downregulated in transcriptome analysis of mycorrhizated and non-mycorrhizated soybean plants (Marquez et al., 2019). *G*. *mosseae* first colonized susceptible maize cultivars (Gaoyou-115 and Yuenong-9) to establish mycorrhizal symbiosis. After successful colonization, the plants were inoculated with *Rhizoctonia solani* to induce infection. The study found that mycorrhizal colonization upregulated the expression of DRGs (e.g., PAL, AOS, and PR2a), enhancing resistance against the pathogen. Additionally, BX9, a gene involved in the biosynthesis of benzoxazinoids (including DIMBOA and related compounds), showed increased expression in the leaves of both cultivars, suggesting a role in systemic defense priming (Ma et al., 2021b). Both nonmycorrhizal genotypes of *Lycopersicon esculentum* (mutant rmc and wild type 76R) infected with *R*. *solani* exhibited similar DRGs expression. However, after inoculation with AMF, the mutant rmc showed increased intracellular mRNA levels of GluBAS and Chi9 and higher extracellular PR-1 expression (Gallou et al., 2011).

### 8.3 AMF-induced defensive enzyme activation in plants

**Figure 3:**
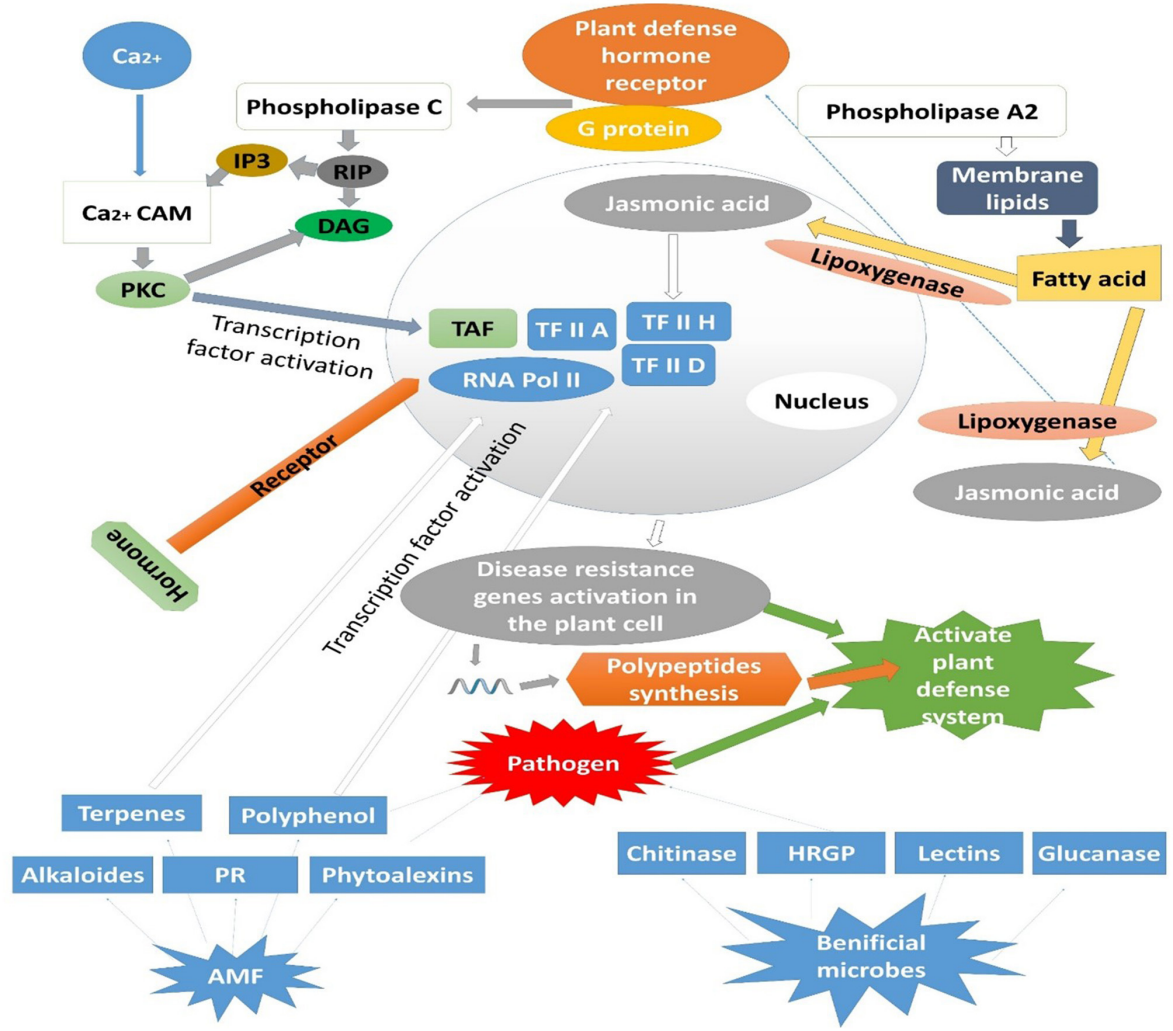
Activation of plant defensive enzymes by pathogen recognition and modulated through colonization of AMF and other beneficial microbes.

AMF initiate defensive enzymes in plants after the development of symbiotic relationships such as PODs and polyphenol oxidase (PPO) (phenolic substance metabolizer), chalcone synthase (CHS) (flavonoid synthesizer), chalcone isomerase (CHI) (metabolizer of lignin, phytoalexin, and isoflavone/flavonoid biosynthesis), phenylalanine ammonia-lyase (PAL) (metabolizer of proteins related to disease resistance (PR proteins) and phenypropanes) (Isayenkov et al., 2005). PAL is a physiological marker of plant resistance to pathogens. PAL activity was enhanced significantly in leaves and stems when infected *F*. *oxysporum* inoculated tomato seedlings were injected with AMF, which resisted disease and infection development (De Román et al., 2010). It was observed that Superoxide dismutase (SOD), 1,1-diphenyl-2-picrylhydrazyl (DPPH), and free radical scavenging activity enhanced in *G. mosy*-infected strawberry plants. The antioxidant production significantly increased in plants, which improved the resistance against *F*. *oxysporum* (Steinkellner et al., 2012). The PR protein, chitinase, glucanase, and other allergic reaction substances are upregulated in mycorrhizated plants with *G*. *mosei*. Inoculation with *G*. *clarum, G*. *monosporum*, and *G*. *deserticola* significantly enhanced the polyphenol oxidase activity in date palms and resisted chlorosis. *G*. *rhizogenes* stimulates the synthesis of *sp7* (a defense protein); in the nucleus, it interacts with *ERF19* (a protein transcription factor relevant to the disease process). The phrase sp7 helps in symptom alleviation induced by *Magnaporthe oryzae* (Kloppholz et al., 2011). AMF can enhance disease resistance in plants, either systemically or locally. The mechanisms by which AMF improves plant resistance may involve a single process or the combined effects of multiple pathways (Tabin et al., 2009). The effectiveness of AMF in suppressing diseases depends on the interactions between viruses, host plants, and AMF, which are influenced by abiotic factors such as soil properties (temperature, moisture, pH, and nutrient levels), timing of inoculation, and inoculum dosage. Additionally, agricultural practices, including farming techniques, pest control strategies, and fertilizer application, play a crucial role in determining the success of AMF-mediated biocontrol in farming ecosystems (Figure 3) (Chandanie et al., 2009).

### 8.4 Transcriptome and proteome profiling of AMF-responsive genes in host plants

Transcriptomic and proteomic profiling are crucial for elucidating the molecular mechanisms underlying plant defense responses influenced by AMF (Aslam et al., 2024). Proteomic analysis facilitates the systematic identification and quantification of proteins expressed in plant roots in response to AMF colonization, offering insights into the functional dynamics of the plant-microbe interaction at the protein level (Yu et al., 2023). RNA of 64-day-old AMF RNA sequencing (RNA-seq) inoculated watermelon plants exhibited 2,259 differentially expressed genes (DEGs) related to signal transduction and metabolic pathways and involved in photosynthesis, nutrient transporters, biosynthesis of chlorophyll, and hormone biosynthesis. Proteomic profiling suggested that AMF is involved in the auxin signaling pathway by triggering auxin response factors, auxin-mediated proteins, auxin transporter-like proteins, and auxin-responsive proteins (Ma et al., 2024). Roots of mycorrhizated wheat plants with *F*. *mosseae* under water scarcity were analyzed for DEGs, 114,428 DEGs were found those involved in N compound, lipid, and carbohydrate metabolic pathways, cellulose synthase, and chitinase activity, and membrane transports and help plants to tolerate water deficit environment (Moradi Tarnabi et al., 2020). RNA-seq exhibited that the mycorrhizated root of snapdragon plants inhibits the osmotic stress induced by cold stress by enhancing the production of proline, soluble sugars, and proteins. Further, AMF attenuated the damage initiated by reactive oxygen spp. through the boost of GSH and AsA contents, PODs, catalase (CAT), SOD, monodehydroascorbate reductase (MDHAR), dehydroascorbate reductase (DHAR), glutathione reductase (GR), and ascorbate peroxidase (APX) activities. Furthermore, proteomic profiling identified AMF involved in regulating genes encoding the photosystem I and II related proteins, phytohormones synthesis, transcription factors related to stress, and active oxygen metabolism (Li et al., 2024b). Transcriptome profiling of mycorrhizated soybean plants with *F. mosseae* and infected with *F*. *oxysporum* showed DEGs (24,285), and genes PAL, CCR, CHI, and CYP73A were found upregulated upon infection with pathogenic fungi and triggered the defense response of soybean. In addition, mycorrhizated soybean roots upregulate of isoflavone metabolic pathway and lead to the synthesis of defense compounds by the production of glycine and daidzein along with substantial changes in the ample amount of terpene and phenolic metabolites, phenolic, and amino acids (Lu et al., 2020).

## 9 Common symbiosis signaling pathway

The common symbiosis-signaling pathway (CSSP) is a conserved signaling cascade that is required for the development of AMF symbiosis and the activation of nutrient exchange between plants and AMF (Maclean et al., 2017). It is activated upon the perception of fungal-derived lipochitooligosaccharides (e.g., Myc factors) and by plant LysM receptor-like kinases, including LYR3 from *Medicago truncatula* (Fliegmann et al., 2016). This interaction induces a downstream signaling cascade involving DMI1, DMI2, DMI3, and CCaMK (Mitra et al., 2004). DMI1 is an ion channel localized in the nucleus that facilitates calcium spiking, a key second messenger in AMF symbiosis (Jiang and Ding, 2023). Patch-clamp electrophysiological experiments revealed that DMI1 played an essential role in the generation of rhythmic calcium oscillations in root epidermal cells after AM fungus recognition (Tian et al., 2020). DMI2, a leucine-rich repeat receptor-like kinase, functions downstream of Myc factor perception and, together with the symbiosis receptor kinase SYMRK, assembles into a complex to activate the CSSP (Zhou et al., 2025a). Phosphoproteomic analysis indicates that DMI2 is quickly auto-phosphorylated in response to fungal signals, thereby activating a phosphorelay cascade that propagates the symbiotic signal (Ivanov and Harrison, 2024). Calcium-activated protein kinase CCaMK, which DMI3 encodes, decodes the calcium-spiking rhythm via calmodulin binding and transcription factor phosphorylation (Dhanker et al., 2020). Structural studies through cryo-electron microscopy (cryo-EM) have also unraveled how calcium-calmodulin binding relieves the auto-inhibitory domain of CCaMK, thereby facilitating downstream transcriptional reprogramming (Yuan et al., 2022). CCaMK also interacts with DELLA proteins, thereby coordinating gibberellin signaling to regulate AM fungal colonization (Yuan et al., 2022). New single-cell RNA-seq data reveal that DMI genes are cell-type-expressed explicitly in the root cortex, where arbuscule formation occurs, during their spatial regulation during symbiosis (Somoza et al., 2024). Epigenetic studies reveal that histone deacetylases regulate DMI expression to maintain proper signaling intensity under different phosphate conditions (Li et al., 2024c). CSSP land plant conservation prioritizes its evolutionary importance as comparative genomics dictates the existence of orthologs in non-legumes, postulating a potential role in promoting AMF symbiosis for sustainable agriculture (Vernie et al., 2025).

## 10 Genomic and pangenomic studies of AMF

The genomic and pangenomic research on AMF has significantly advanced our understanding of their evolutionary biology, symbiosis, and functional diversity (Liu and Chen, 2024). The genomic sequencing of the fungus *Rhizophagus irregularis* (genome accession no.: DAOM-197198) provided the first complete genome of an AMF (Masclaux et al., 2019). This analysis exhibited a diminished suite of plant cell wall-degrading enzymes with an expanded suite of symbiotic signaling genes, including those in the common symbiosis pathway (Tisserant et al., 2013). Pangenomic analyses of *Rhizophagus* and *Gigaspora* spp. have likewise revealed significant genomic plasticity, in which strain-specific genes are associated with host adaptation and nutrient exchange (Oliveira et al., 2024). The research uncovered evidence of horizontal gene transfer from bacterial origins, which accounts for the metabolic versatility of AMF (Li et al., 2018). Pangenomic approaches unveiled the core and accessory genomes, with a focus on the contribution of transposable elements to genomic development (He et al., 2024). The findings emphasize the need for more extensive sampling approaches to achieve the genomic diversity of AMF and inform subsequent research on their ecological and agricultural significance.

## 11 Conclusion

This comprehensive review underscores the pivotal role of AMF in sustainable crop disease management and yield enhancement. AMF establishes intricate symbiotic associations with plant roots that greatly boost nutrient uptake, water absorption, and protection from biotic and abiotic stresses. Besides their conventional role as a facilitator of nutrient mobilization, AMF induces SR through hormonal signaling, upregulation of DRGs, and SMs biosynthesis. Their symbiotic and non-symbiotic interactions with beneficial rhizosphere microbiota also enhance their biocontrol activity against a range of phytopathogens. AMF-mediated root exudate alteration, porosity of soil, and structure of microbial community create a suppressive soil status that is unfavorable for pathogens. Molecular studies, including proteomics and transcriptomics, have explained the potential of AMF in modulating host plant immunity at biochemical and genetic levels. Most generally, AMF offers a promising, sustainable alternative to chemical inputs to modern agriculture that is consistent with global sustainability and food security goals.

## 12 Future aspects

Future research must endeavor to optimize AMF inoculants for diverse agroecosystems through the identification of host-specific strains and environmental tolerance. Metagenomics and transcriptomics can resolve tripartite interactions among AMF, plants, and pathogens. Field trials with varying climatic and soil conditions will validate efficacy, while precision agriculture tools can integrate AMF for pinpoint delivery. Investigation of synergistic effects from interaction with other biocontrol agents (e.g., PGPR, *Trichoderma*) through combinatorial testing will enhance disease control measures. Long-term studies on soil fertility and carbon sequestration through glomalin production are a must. Lastly, the scaling up of AMF production processes will enable commercial viability for sustainable agriculture.
